# *VTC4* Polyphosphate Polymerase Knockout Increases Stress Resistance of *Saccharomyces cerevisiae* Cells

**DOI:** 10.3390/biology10060487

**Published:** 2021-05-30

**Authors:** Alexander Tomashevsky, Ekaterina Kulakovskaya, Ludmila Trilisenko, Ivan V. Kulakovskiy, Tatiana Kulakovskaya, Alexey Fedorov, Mikhail Eldarov

**Affiliations:** 1 Federal Scientific Center, Pushchino Research Center for Biology of the Russian Academy of Sciences, Skryabin Institute of Biochemistry and Physiology of Microorganisms, Russian Academy of Sciences, pr. Nauki 5, 142290 Pushchino, Russia; tomashevskialexandr25@gmail.com (A.T.); ekaterina.kulakovskaya@gmail.com (E.K.); tril2020tril@rambler.ru (L.T.); 2 Center for Precision Genome Editing and Genetic Technologies for Biomedicine, Engelhardt Institute of Molecular Biology, Russian Academy of Sciences, 119991 Moscow, Russia; ivan.kulakovskiy@gmail.com; 3 Vavilov Institute of General Genetics, Russian Academy of Sciences, 119991 Moscow, Russia; 4 Federal Scientific Center for Biotechnology of the Russian Academy of Sciences, Institute of Bioengineering, Russian Academy of Sciences, Leninsky prosp. 33-2, 119071 Moscow, Russia; anfedorov@yahoo.com (A.F.); eldarov1@yandex.ru (M.E.)

**Keywords:** inorganic polyphosphate, yeast, *VTC4*, oxidative and heavy metal stress, *DDR2*, *PHO84*

## Abstract

**Simple Summary:**

Inorganic polyphosphate, a linear polymer of orthophosphoric acid, plays an important role in microorganisms’ stress resistance. Vtc4 protein synthetizes inorganic polyphosphate in yeast. Here we show that yeast lacking this protein exhibit very low polyphosphate level, decreased resistance to alkaline stress, but increased resistance to oxidative and heavy metal stresses. We suggest the increased stress resistance is achieved by constitutive up-regulation of oxidative stress-response genes and decreased expression of Pho84 that is responsible for manganese uptake.

**Abstract:**

Inorganic polyphosphate (polyP) is an important factor of alkaline, heavy metal, and oxidative stress resistance in microbial cells. In yeast, polyP is synthesized by Vtc4, a subunit of the vacuole transporter chaperone complex. Here, we report reduced but reliably detectable amounts of acid-soluble and acid-insoluble polyPs in the Δ*vtc4* strain of *Saccharomyces cerevisiae*, reaching 10% and 20% of the respective levels of the wild-type strain. The Δ*vtc4* strain has decreased resistance to alkaline stress but, unexpectedly, increased resistance to oxidation and heavy metal excess. We suggest that increased resistance is achieved through elevated expression of *DDR2*, which is implicated in stress response, and reduced expression of *PHO84* encoding a phosphate and divalent metal transporter. The decreased Mg^2+^-dependent phosphate accumulation in Δ*vtc4* cells is consistent with reduced expression of *PHO84*. We discuss a possible role that polyP level plays in cellular signaling of stress response mobilization in yeast.

## 1. Introduction

Inorganic polyphosphate (polyP), the linear polymer of orthophosphoric acid, is a universal regulatory biopolymer in living cells [1,2,3,4]. PolyP and enzymes of its metabolism are involved in various processes regulating vital activities of the cell. In bacteria, PolyPs are important for stress response and virulence [1,5,6], whereas, in the human organism, polyPs play is involved in bone tissue growth and development [7,8], blood coagulation cascade, inflammatory response [9], and signal transduction in astrocytes [10]. Furthermore, PolyP is a component of a specific calcium channel in mitochondrial membranes regulating calcium level and stress response [11].

In yeast, polyPs are involved in the control of the cell cycle [12], stress response [13,14,15], and virulence [16]. Yeast genomes do not contain orthologs of bacterial polyphosphate kinases, and polyP synthesis is performed by the Vtc4 protein [17]. Vtc4 is a part of the Vacuole Transporter Chaperone (VTC) complex, which plays an important role in vacuolar membrane fusion and has physical relations with vacuolar H^+^-ATPase (V-ATPase), influencing vacuolar H^+^ uptake [18,19]. In *Saccharomyces cerevisiae,* the VTC complex consists of five subunits: Vtc1, Vtc2, Vtc3, Vtc4, [18,19], and Vtc5 [20]. The search for homologues of VTC complex proteins in fungi revealed that the complex is ancient and at least one component (Vtc4, but likely also Vtc1) was present early in the evolutionary history of fungi, while Vtc2 and Vtc3 result from a recent duplication in the *S. cerevisiae* lineage [21].

The mechanism of Vtc4 polyphosphate polymerase activity has been clarified using X-ray crystallography, which has revealed that the Vtc4 fragment contains a long-chain electron-dense domain winding through the tunnel, suggesting that this module generates polyPs from ATP [17]; the catalytic domain faces the cytoplasm and the polymer must pass through the membrane. This Vtc4 fragment demonstrated phosphotransferase activity and could synthesize polyPs in solution from ATP, releasing ADP in the presence of Mn^2+^.

The *vtc4* null mutants of *S. cerevisiae* [22,23] and *Cryptococcus neoformans* [16] have significantly reduced polyP levels. The Δ*vtc4* deletion strains of *S. cerevisiae* lack the entire vacuolar polyP pool; Δ*vtc1* point mutations targeting the conserved basic residues of transmembrane domains also reduce cellular polyP level [17]. Vacuoles from the cells of Δ*vtc1* strain do not synthesize polyP in vitro [23]. The decrease in polyP level was also observed in *S. cerevisiae* null mutants in *VTC2*, *VTC3* [22]. It has been proposed that the small, membrane-integral Vtc1, together with the transmembrane domains of Vtc2 and Vtc3 proteins, forms a channel that transfers polyP into the organelle lumen [24].

The VTC in the cells of *S. cerevisiae* exists as two sub-complexes: Vtc4/Vtc3/Vtc1 and Vtc4/Vtc2/Vtc1; the first is located mostly in the vacuole membrane, and the second can also be observed in the endoplasmic reticulum, nuclear envelope and, under phosphate starvation conditions, in the vacuolar membrane [24]. The two differently regulated sub-complexes possibly create polyP pools with different functions. Vtc2, Vtc3, and Vtc4 contain the SPX domain that provides a binding surface for inositol phosphate signaling molecules, whose concentrations change depending on the availability of inorganic phosphate (Pi) [24,25,26]. The search of SPX domains in other proteins resulted in the detection of one more component of the VTC complex: the Vtc5 subunit [20]. Vtc5 regulates polyP synthesis and phosphate homeostasis in yeast [20]. This protein physically interacts with VTC, and deletion in the respective gene decreases the polyP level, while overexpression results in increased polyP level both in vivo and in isolated vacuoles [20].

Obviously, the functioning of the VTC complex depends on phosphate availability and the phosphate signal transduction (PHO) pathway. Yeast cells possess two low-affinity H^+^/Pi symporters Pho87 and Pho90, high-affinity H^+^/Pi symporter Pho84 and high-affinity Na^+^/Pi symporter Pho89 in the plasma membrane [27,28]. Low-affinity phosphate/sodium symporter Pho91 is localized in the vacuole membrane and is essential for the storage and mobilization of vacuolar polyP [29]. The PHO pathway in yeast consists of the PHO-specific regulatory proteins Pho2 and Pho4 (the transcriptional activators), Pho80–Pho85 (the cyclin-dependent kinase complex), and the cyclin-dependent kinase inhibitor Pho81 [30,31]. When cells are Pi-starved, Pho81 inhibits the activity of the Pho80–Pho85 complex, which phosphorylates Pho4. The non-phosphorylated Pho4 is localized in the nucleus and activates the transcription of target genes with the Pho2 transcription cofactor. The PHO pathway induces the expression of genes that encode high-affinity transport proteins (Pho84 and Pho89) and genes that encode secreted acid phosphatases (Pho5, Pho11 and Pho12) [30,31]. More than 300 genes are involved in the regulation of the PHO pathway in yeast [32]. The mechanisms of interaction between cellular polyP and the PHO pathway are complex. The correlation of *PHO5* expression with the levels of both intracellular orthophosphate and intracellular polyP was demonstrated [33]. The proteins of the VTC complex, phosphate transporters Pho87, Pho90, and Pho91, and Pho81 contain SPX domains, indicating their regulation by inositol pyrophosphates [34].

Vtc4 is the main enzyme that performs polyP synthesis in fungi. Almost zero polyP level in the Δ*vtc4* strain of *S. cerevisiae* was demonstrated by several extraction methods [17,22,23]. PolyP were undetectable by NRM in Δ*vtc4* cells in vivo [33,35,36]. However, in some fungi species, polyP was detectable in Δ*vtc4* mutants: a limited amount of polyP was extracted from Δ*vtc4* cells of *Ustilago maydis* and was attributed to phosphate storage in the nucleus, mitochondrion, or cell wall [37].

The cells of *S. cerevisiae* contain several polyP pools, which differ in chain length and subcellular localization [38,39,40]. The NRM assay in vivo reveals presumably vacuolar polyP [41]. To verify the presence of polyP in Δ*vtc4* mutants of *S. cerevisiae*, in this study, we comparatively assessed individual polyP fractions, including salt- and alkali-soluble polyPs, in Δ*vtc4* and the wild-type strain. The Δ*vtc4* mutants with reduced polyP pool provide a suitable model to investigate the role of polyP in overcoming stress [41,42,43]. Here, we report the effect of *VTC4* knock out on stress resistance of *S. cerevisiae*.

## 2. Materials and Methods

### 2.1. Yeast Strains and Growth Conditions

The *S. cerevisiae* wild-type (WT) strain YSC-1048, the Δ*vtc4* mutant, and the Δ*pho84* mutant were obtained from the Dharmacon collection. In the experiments with Pi uptake, we used CRN strain (MATa ade2 his3 ura3 ppn1Δ::CgTRP1, kindly provided by A. Kornberg [44]) and PPN1 polyphosphatase-overexpressing CRN/PPN1 (MATa ade2 his3 ura3 ppn1Δ:CgTRP1 transformed with pMB1/PPN1 Sc) [45]. The Ppn1-overexpressing strain (CRN/PPN1) was constructed from the parent CRN strain by transfection with the pMB1 expression vector, which contained an expression cassette with the strong constitutive *TDH3* promoter and *PGK* terminator [45]. Cells were cultured in YPD medium containing 2% glucose, 2% peptone (Sigma-Aldrich, St. Louis, MO, USA), and 1% yeast extract (Pronadisa, Madrid, Spain) at 29 °C and 145 rpm until the stationary growth stage, harvested by centrifugation at 5000× *g* for 10 min and washed twice with sterile distilled water.

### 2.2. PolyP Extraction and Measurement

The polyPs were extracted as described earlier [38,39] with minor modifications. To obtain an acid-soluble polyP fraction (polyP1), the yeast cell biomass was treated twice with 0.5 M HClO_4_ at 0 °C for 20 min with stirring. After the separation of the supernatant, the remaining biomass was treated twice with saturated NaClO_4_ solution at 0 °C for 30 min; the supernatant collected after centrifugation represented a salt-soluble polyP fraction (polyP2). Then, a weak alkali-soluble fraction (polyP3) and an alkali-soluble fraction (polyP4) were extracted with 0.1 mM NaOH (pH 10) or 50 mM NaOH, respectively, at 0 °C for 30 min. The rest biomass was incubated in 0.5 M HClO_4_ at 90 °C for 20 min, and the released Pi was attributed as polyP5 fraction. The impurities of nucleic acids and nucleosides were removed from extracts by activated charcoal [46]. The extracts were treated with activated charcoal Norit (Sigma-Aldrich) (0.1 g per 1 mL of each extract) for 30 min at 0℃ with shaking. The charcoal was removed by filtration, and absorption values at 260 nm were measured in a 1 cm cuvette.

The polyPs in the obtained fractions were assayed as acid-labile phosphorus, i.e., Pi released after treatment with 0.5 M HCl at 90 ℃ for 20 min [38,39]. Pi was measured in all samples before this treatment. The difference between the Pi amount after and before hydrolysis was considered as polyP [38,39]. The last fraction (polyP5) was characterized by the amount of Pi produced after biomass hydrolysis in 0.5 M HClO_4_ at 90 °C for 20 min. The Pi amount was measured as described earlier [47] using an EFOS 9505 photometer (Sapphire, Moscow, Russia).

### 2.3. Enzymatic Assay of PolyPs

For the enzymatic assay, the samples of polyP fractions polyP1, polyP2, and polyP3 were neutralized to pH 7.0 by HCl or NaOH aliquots and incubated with *S. cerevisiae* exopolyphosphatase Ppx1 obtained as described earlier [48]. The reaction mixture containing 0.5 mL of 50 mM Tris-HCl (pH 7.2), 2.5 mM MgSO_4_, 0.02 mL (~5 U) of Ppx1 preparation, and 0.1 mL of polyP extracts was incubated at 30 °C for 2 h with shaking, and the released Pi was assayed as previously described [47]. Commercial polyP_188_ (Monsanto, Creve Coeur, MO, USA) was used as control.

### 2.4. PolyP Electrophoresis

The preparations of polyP fractions polyP1, polyP2, and polyP3 (See Section 2.2) were precipitated from extracts with a saturated solution of Ba(NO_3_)_2_, pH 8.2, and re-solved by treating with Dowex AG 50Wx8 (Sigma-Aldrich) ion exchange resin in (NH_4_)^+^ form [38]. The samples were treated with enzyme preparations in 50 mM Tris-HCl pH 7.0 for 1 h at 30 ℃. The incubation mixture contained 5 mM of MgSO_4_ in the cases of the treatment with Ppx1 and DNase. Ppx1 (~5 U) [48], RNase A (0.2 mg/mL) (Sigma-Aldrich, St. Louis, MO, USA) and DNase ColE 9 (0.2 mg/mL) were used in these experiments. The DNase ColE 9 was obtained as described earlier [49] and kindly provided by Dr. I. Granovsky (IBPM RAS, Pushchino, Russia). The polyP samples incubated without enzymes in the same conditions in the presence and absence of MgSO_4_ were used as the control.

The chain length of polyPs was assessed by electrophoresis as described previously [50] in 24% polyacrylamide gels with 7 M urea; commercial polyP_15_, polyP_25_, and polyP_75_ (Sigma-Aldrich) were used as standards (the numbers indicate the average amount of phosphate residues in the polyP chain). PolyPs were detected by staining the gels with the toluidine blue.

### 2.5. Determination of Yeast Sensitivity to Peroxide, Alkali, and Heavy Metal Ions

Yeast samples normalized by cell concentration (0.5 × 10^7^ cell/ mL) were added to the wells of sterile plates containing the YPD medium and different concentrations of Cd(CH_3_COO)_2_⋅2H_2_O, MnSO_4_, CoSO_4_, NiSO_4_, hydrogen peroxide, or KOH. The cells were cultured for 24 h, and the optical density was measured at 594 nm using an EFOS photometer.

### 2.6. Pi Accumulation

The cells were cultivated in YPD or in Pi limited YPD for the stationary stage. The Pi limited YPD was prepared according to Rubin [51]. Freshly harvested yeast cells (~55 mg wet biomass) were incubated in 0.75 mL of MiliQ water containing 110 mM glucose and 1 mM K_2_HPO_4_ and supplemented or not with 5 mM MgSO_4_, at 30 °C with shaking (850 rpm) in ThermoMixer (Eppendorf, Hamburg, Germany). After 45 min, the cells were centrifuged at 5000× *g*, and Pi was measured in supernatants by the colorimetric method with malachite green [48].

### 2.7. Quantitative PCR

The yeast cells were grown in 25 mL of YPD medium in 250-mL flasks at 28 °C, and biomass from 10 mL of culture was harvested after 36-h growth (stationary phase). Total RNA was extracted using the acid hot phenol method [52]; two biological replicates were performed. RNA quality was assessed by electrophoresis in 1.5% agarose TBE gels. After the treatment of RNA with DNase I (Thermo Fisher Scientific, Waltham, MA, USA) followed by purification using the RNA CleanUp Kit (Evrogen, Moscow, Russia), the cDNA was synthesized using the cDNA RevertAid First Strand cDNA Synthesis Kit (Thermo Fisher Scientific, Waltham, MA, USA) and random hexamer primers.

The qPCR was carried out in a CFX96 Cycler-RealTime PCR Detection System (Bio-Rad Laboratories, Inc., Hercules, CA, USA) using SYBR Green 2,5 Master Mix (Syntol, Moscow, Russia) and *S. cerevisiae* gene-specific primers (Supplemental Table S1) designed with Primer-BLAST (http://www.ncbi.nlm.nih.gov/tools/primerblast/, accessed on 1 September 2020) and synthesized by Evrogen (Moscow, Russia). The reactions were performed with 2.5 ng of cDNA at the following cycling conditions: initial denaturation at 95 °C for 5 min and 40 cycles of denaturation at 95 °C for 15 s and annealing/extension at 60 °C for 40 s. To normalize gene expression levels, the *S. cerevisiae ALG9* gene was used as a reference [53]. The qPCR results were statistically analyzed with Graph Pad Prism version 7.02 (GraphPad Software Inc., San Diego, CA, USA; https://www.graphpad.com/scientific-software/prism/, accessed on 1 September 2020) and gene expression levels were calculated relative to *ALG9* expression using the 2^−ΔΔCT^ method [54].

### 2.8. Statistics

The experiments were performed in no less than3 replicates, except the electrophoresis experiment that was repeated twice. Statistical analyses were performed in R software using the Student’s *t*-test.

## 3. Results

### 3.1. VTC4 Knockout Strain Has Decreased but Detectable PolyP Level

As there is no standard method of polyP extraction in yeast, we applied a reliable multi-stage extraction protocol [38] that provides the most complete extraction of polyP from yeast cells [41] and allows obtaining separate fractions of polyPs with different chain length [38,39]. Next, we characterized the fractions in terms of their chain length, nucleic acids contamination, and Ppx1 hydrolysis.

**Figure 1:**
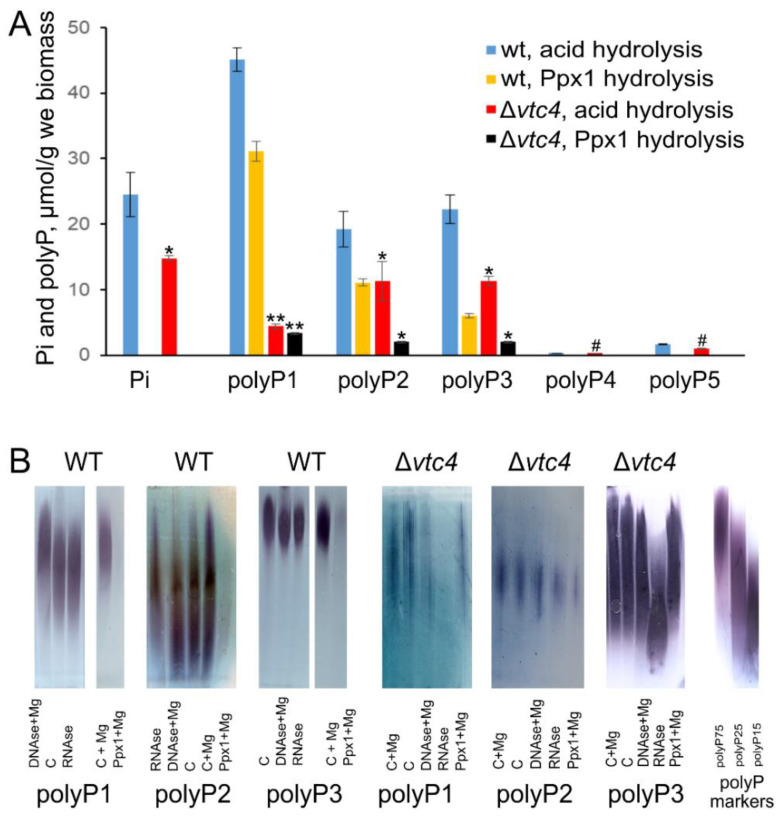
Pi and polyP in WT and Δ*vtc4 S. cerevisiae* strains. (**A**) The amount of Pi and polyPs in different fractions measured by acid hydrolysis and Ppx1 hydrolysis. The experiments were performed in 3 replicates, the values denote mean, and the error bars denote s.d. ** *p* < 10^−3^, * *p* < 0.05, #—n.s., significance was assessed with the one-tailed Student’s *t*-test performed for Δ*vtc4* against WT, the results of acid hydrolysis and Ppx1 hydrolysis were assessed separately; (**B**) A representative electropherogram of polyP1, polyP2, and polyP3 fractions of WT and Δ*vtc4 S. cerevisiae* strains. C—control treatment without enzymes. PolyP markers were commercial polyP (Sigma, USA) with an average chain length of 75 (polyP75), 25 (polyP25), and 15 (polyP15) phosphate residues. The experiment was repeated twice.

The Pi level in the Δ*vtc4* strain was lower than that in the WT strain (Figure 1A). The amount of polyP in polyP4 and polyP5 fractions was comparably small in both strains (Figure 1A). The amounts of polyP in these fractions were insufficient for enzymatic and electrophoretic determination in both strains. PolyPs in polyP1, polyP2, and polyP3 fractions were hydrolyzed by Ppx1 polyphosphatase; however, in both strains, the hydrolysis was incomplete (Figure 1A). Partial inaccessibility of polyPs from biological sources for enzymatic hydrolysis has been reported earlier [44,55], but the reason is still unclear. One explanation can be the presence of Ca^2+^ or Fe^2+^, which inhibit Ppx1 activity [56]. The RNA impurities and low polyP concentration may explain the decreased level of polyP hydrolysis by Ppx1 in the case of polyP preparations from the cells of the Δ*vtc4* strain.

Electrophoregrams revealed characteristic bands stained by toluidine blue in polyP1, polyP2, and polyP3 fractions (Figure 1B). The weak effect of Ppx1 treatment in the case of Δ*vtc4* mutant agreed with incomplete hydrolysis by Ppx1. Of note, the A_260_ of polyP1–polyP5 extracts did not exceed 0.05–0.1, indicating a low level of contamination with nucleic acids and nucleosides. However, in the case of polyP preparations from Δ*vtc4*, the pretreatment of samples with barium salts probably increased the contamination with nucleic acids. Indeed, the electrophoresis showed that the preparations from the cells of Δ*vtc4* mutant contained RNA.

However, Ppx1 is highly specific for polyP, and the RNA contamination cannot lead to the overestimation of Pi released by the enzyme. The enzymatic assay provided the following estimates of the polyP content (Figure 1A): the cells of Δ*vtc4* mutant contained 10% and 20% of acid-soluble polyP and 20% of acid-insoluble polyP of the respective amounts in the parent strain. The total polyP content determined by enzymatic assay with Ppx1 was 48 and 7.4 μmol P/g wet biomass for WT and Δ*vtc4* strains, respectively.

### 3.2. The *Δ*vtc4 Strain Is More Sensitive to Alkali but Resistant to Peroxide and Heavy Metals

**Figure 2:**
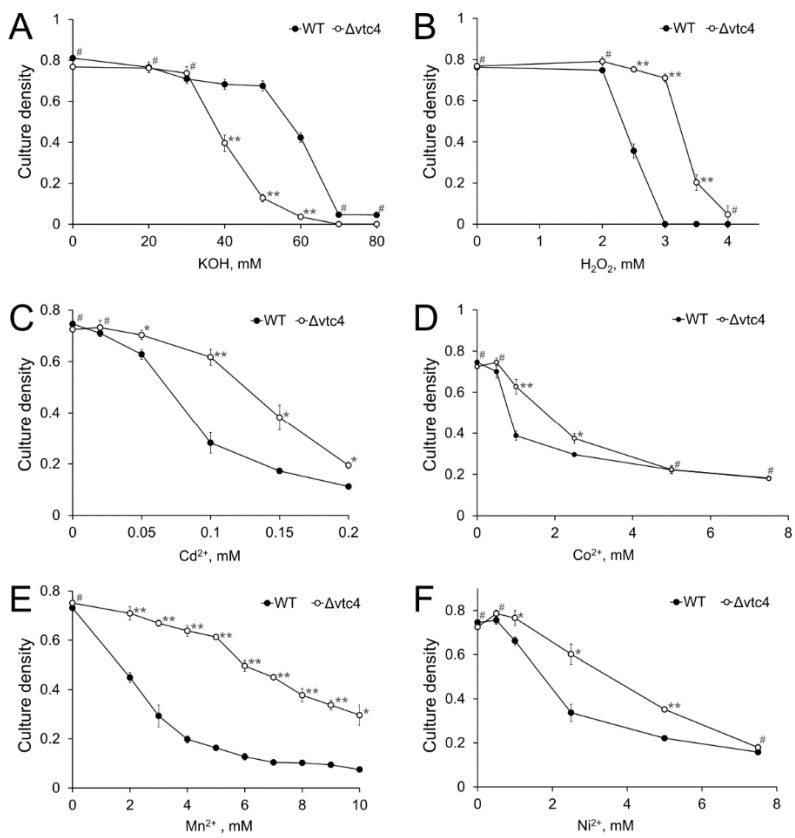
The effects of alkali (**A**), hydrogen peroxide (**B**), cadmium (**C**), cobalt (**D**), manganese (**E**), and nickel (**F**) on the growth of WT and Δ*vtc4 S. cerevisiae* strains. The experiments were performed in 4 replicates, the values denote mean culture density, and the error bars denote s.d. ** *p* < 10^−4^, * *p* < 0.05, #—n.s., significance was assessed with the 2-tailed Student’s *t*-test performed for Δ*vtc4* against WT at the same concentration of effectors (*x*-axes).

Vtc4 is involved in the function of vacuoles [16,17], which play a significant role in the stress resistance of yeast [57]. We compared the sensitivity of WT and Δ*vtc4* strains to high pH, hydrogen peroxide, and heavy metals. The Δ*vtc4* strain was more sensitive to alkaline stress, as evidenced by total growth cessation in the presence of 60 mM KOH, whereas the growth of the WT strain at this concentration was inhibited only by 50% (Figure 2A). Unexpectedly, the Δ*vtc4* strain was more resistant to the other stresses (Figure 2B–F). The most pronounced difference between the strains was observed in the presence of Mn^2+^. The growth of the WT strain was markedly inhibited at 2 mM MnSO_4_, whereas for the Δ*vtc4* strain, the same level of growth inhibition was observed at 7 mM MnSO_4_ (Figure 2E). However, the excess of other heavy metals and hydrogen peroxide also lead to significant differences in growth between WT and Δ*vtc4* strain (Figure 2B–D,F).

### 3.3. Differential Expression of Selected Genes Allows to Explain Stress Resistance of *Δ*vtc4 Cells

The resistance to manganese and peroxide stresses observed in the Δ*vtc4* strain was also observed for the Ppn1-overexpressing CRN/PPN1 strain, which also had decreased polyP level [14]. The CRN/PPN1 strain showed constitutively higher expression of genes associated with response to external stimulus, plasma membrane organization, and oxidation/reduction. Manganese resistance of the CRN/PPN1 strain was associated with the downregulation of *PHO84*. We consider the Δ*vtc4* strain could have a molecular phenotype partly similar to that of the CRN/PPN1 strain, even in non-stress conditions.

**Figure 3:**
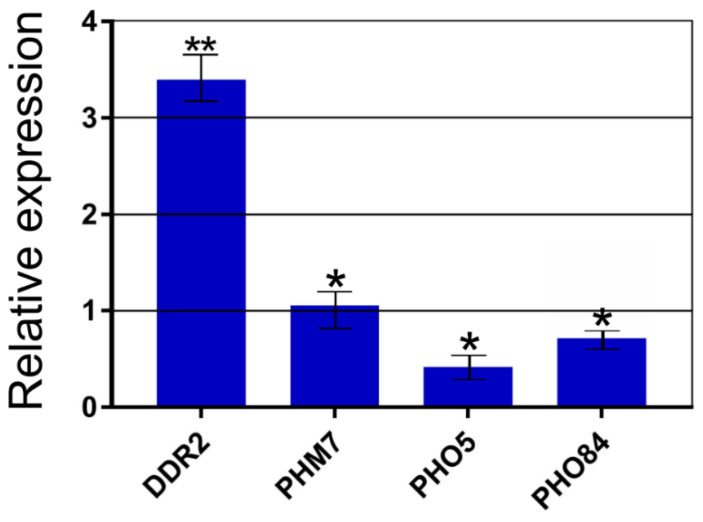
Differential expression of the selected target genes between the Δ*vtc4* and WT strains. Y-axis: the relative mRNA abundance (Δ*vtc4* normalized by WT) estimated by qPCR. The experiments were performed in 3 replicates, the values denote mean, and the error bars denote s.d. ** *p* < 0.01, * *p* < 0.05, significance of difference between Δ*vtc4* and WT was assessed with a 2-tailed Student’s *t*-test.

We selected several genes that were differentially expressed in the CRN/PPN1 strain (compared to the parent CRN strain). Next, we estimated their relative expression in Δ*vtc4* versus the WT strain (Figure 3). Among those genes, there was *DDR2* thatencodes the multi-stress response factor activated by a variety of xenobiotic agents and environmental or physiological stresses [58]. Similar to the CRN/PPN1 strain, the *DDR2* gene was strongly upregulated in the Δ*vtc4* strain (with a three-fold higher expression compared to the WT, Figure 3).

The expression of the *PHM7* gene, which encodes a putative transport protein [22], was upregulated in the CRN/PPN1 strain, but it did not differ between the Δ*vtc4* and WT strains. The *PHO5* and *PHO84* genes encoding an acid phosphatase and Pi transporter Pho84, respectively, were downregulated, although, in the case of Pho84, the effect was less pronounced; the expression of these two genes also decreased in the CRN/PPN1 strain.

### 3.4. *Δ*vtc4 Cells Show Decreased Pi Accumulation

The high-affinity phosphate transporter Pho84 of *S. cerevisiae* is involved in the uptake of both Pi and divalent metal ions [59,60]. Therefore, we expected a decrease in the magnesium-stimulated accumulation of phosphate by the cells of both strains with decreased *PHO84* expression, CRN/PPN1 [14] and Δ*vtc4*.

**Figure 4:**
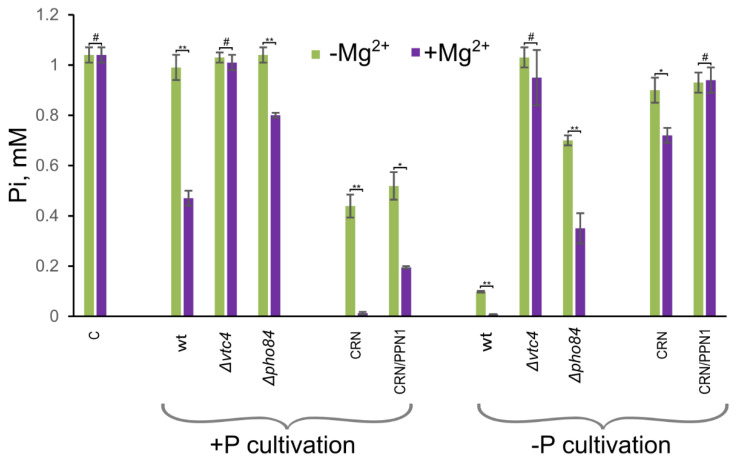
The Pi concentration in the medium after incubation with the cells of *S. cerevisiae*. The stationary grown cells of WT, Δ*vtc4*, Δpho84, CRN, and CRN/PPN1 strains were incubated in water containing 110 mM glucose and 1 mM K_2_HPO_4_ and supplemented or not with 5 mM MgSO_4_. +P cultivation—the cells were pre-cultivated in the YPD medium with 4 mM Pi; −P cultivation—the cells were pre-cultivated in the YPD medium with 0.05 mM Pi; C—the medium was incubated without the cells, and the Pi concentration was measured with the same assay method. The experiments were performed in 3 replicates, the values denote mean, and the error bars denote s.d. ** *p* < 0.001, * *p* < 0.05, #—n.s., significance was assessed with the 2-tailed Student’s *t*-test comparing Pi concentrations (as shown by brackets) in the presence (+) and absence (-) of Mg^2+^.

We compared Pi accumulation in the mutant strains with that in the respective control (WT and CRN) strains. For the experiments, we used model non-growth conditions and measured the decrease in Pi content in the medium after incubation with the yeast cells [61]. Figure 4 shows concentrations of Pi in the medium after incubation with the cells of different strains. The lower this concentration is, the more Pi is absorbed by the cells. Cells of the Δ*vtc4* and CRN/PPN1 strains showed weaker magnesium stimulation of Pi accumulation compared with WT and CRN strains, respectively.

The Δ*pho84* strain used as control also showed poor magnesium stimulation of Pi accumulation. This effect was observed in cells pre-cultivated in Pi rich as well as Pi limited media. These results are consistent with the downregulation of *PHO84* in the CRN/PPN1 [14] and Δ*vtc4* strains. The decrease in phosphate accumulation capacity in the Δ*vtc4* strain was even more pronounced than in the Δ*pho84* strain. This means that the lowered expression of *PHO84* is not the only cause of the reduced Pi accumulation, i.e., there should be other phosphate transporters that are suppressed in the Δ*vtc4* mutant.

## 4. Discussion

The VTC complex has not been found in mammalian cells [4], and the search of alternative pathways for polyP biosynthesis in yeast may be useful for mapping the polyP biosynthesis pathways in higher eukaryotes. The alternative enzymatic activities resulting in polyP synthesis, such as dolichyl diphosphate: polyP phosphotransferase (EC 2.7.4.20) [62] and 3-phospho-D-glyceroyl-phosphate:polyphosphate phosphotransferase (EC 2.7.4.17) [63] was observed in fungi, but the respective enzymes were not identified yet. The Δ*vtc4* mutant should provide a convenient model to identify such enzymes and their cellular localization.

The Δ*vtc4* strain has several surprising features. First, despite the lack of the primary polyP biosynthesis enzyme, it has a detectable polyP level. Probably, these polyPs belong to special fractions; for example, they could form complexes with polyhydroxybutyrate (PHB) in membranes [64] or belong to lysine-residues of polyP-phosphorylated proteins [43].

Second, Δ*vtc4* shows increased resistance to oxidation and heavy metal stress. In this respect, the Δ*vtc4* mutant is similar to the Ppn1-overexpressing strain, which, as Δ*vtc4* strain, also has reduced polyP level [14]. Of note, reduced resistance of Δ*vtc4* to the alkaline stress is consistent with the polyPs role as a buffer against alkali [65].

The Ppn1-overexpressing strain was previously reported to have stress resistance capabilities similar to those observed for Δ*vtc4* [14]. This motivated to verify possible similarities in differential expression of key genes. On the one hand, the Ppn1-overexpressing strain did not exhibit expression changes of *SMF1/SMF2*, *PMR1*, and *CCC1* that are responsible for manganese detoxification. On the other hand, there were differentially expressed genes related to stress response (*DDR2*) and phosphate uptake (*PHO84*). The upregulation of *DDR2* and downregulation of *PHO84* were consistent between the Ppn1-overexpressing and Δ*vtc4* strain (when compared against respective controls). Of note, DDR2 is not essential for stress response to heat shock and DNA damage [58]. We do not have evidence of its direct role in resistance to oxidative or heavy metal stress, but given its consistently increased expression, we consider this gene as a useful marker of a pre-adapted state.

The manganese resistance and decrease of cellular manganese in the Δ*vtc4* strain were observed earlier [60]. It was suggested that loss of polyP synthesis in Δ*vtc4* mutants serves as a signal to inhibit the uptake of phosphate or manganese phosphate complexes by Pho84 [60]. Pho84 is a part of the PHO pathway in yeast regulating Pi homeostasis and other cellular functions [36]. The reduced expression of Pho84 was observed in the Δ*vtc5* cells, which are defective in polyP accumulation [20]. The de-regulation of *VTC5* expression alters the activation of the PHO pathway [20]. The cells of Δ*vtc5* strain contain the transcription factor Pho4 in the cytosol, such as cells on a Pi-rich medium, whereas cells overexpressing *VTC5* contain Pho4 in the nucleus and mimic Pi-starving cells [20]. The link between polyP accumulation and the PHO pathway is supported by several studies [20,33,34]. We speculate that short-chain polyPs serve as signaling molecules, and their decrease leads to the activation of stress response genes. This effect can be associated with the antioxidant properties of polyPs [66]. Probably, inositol pyrophosphates are involved in this signaling: the Ddp1 enzyme hydrolyzes both polyP and inositol pyrophosphates [67], so the decrease in polyP level can activate the hydrolysis of inositol pyrophosphates.

## 5. Conclusions

Cells of the Δ*vtc4* strain contain only up to 15% of polyP amount typical for wild-type cells. The Δ*vtc4* strain has decreased resistance to alkaline stress and increased resistance to oxidative and heavy metal stresses. The increased resistance of the Δ*vtc4* strain is achieved by the activation of stress-response genes and reduced expression of Pho84. In this regard, the Δ*vtc4* strain is similar to the Ppn1-overexpressing strain, which also has constitutively reduced polyP level. We consider the decreased polyP level as the signal for the activation of selected stress-related genes and downregulation of *PHO84* expression.

## Data Availability

The raw data are available on request.

## Supplementary Materials

The following are available online at https://www.mdpi.com/article/10.3390/biology10060487/s1: Supplemental Table S1: *S. cerevisiae* gene-specific primers used in quantitative real-time qPCR experiment.
