# Supplementary material for: VTC4 Polyphosphate Polymerase Knockout Increases Stress Resistance of Saccharomyces cerevisiae Cells
Source: Biology (Basel). 2021 May 30;10(6):487. doi: 10.3390/biology10060487 (PMC8227513; doi:10.3390/biology10060487)
Supplement: Supplementary file 1 [file biology-10-00487-s001.zip › biology-1223663-supplementary.pdf]

Supplemental Table S1. *S. cerevisiae* gene-specific primers used in quantitative real-time qPCR experiment.

Gene Standard Name	Gene Systematic Name	Gene Brief description	Primer	Oligonucleotide sequence (5′ > 3′)
ALG9	YNL219C	Mannosyltransferase, involved in N-linked glycosylation	Alg9_F	TAC CGG TAA AGA GTG GCC GA
			Alg9_R	TGC AGC ACA TGC CAG CTT AT
DDR2	YOL052C-A	Multi-stress response protein	Ddr2_F	AAA AAG GCC AAA GCA CCA CC
			Ddr2_R	AAA ACG CAT CCA ACA CCA CG
PHM7	YOL084W	Protein of unknown function	Phm7_F	TCT TTT GGG CTT TCC CCG TT
			Phm7_R	GAC ACC CAT CAG GAA GGT GG
PHO5	YBR093C	Repressible acid phosphatase	Pho5_F	TCA ACA TCA CCT TGC AGA CTG T
			Pho5_R	TCG TAG TCC CAA GCA GGA CA
PHO84	YML123C	High-affinity inorganic phosphate (Pi) transporter	Pho84_F	ACG CAA ACT CTG GTG CTG AA
			Pho84_R	CCA AAA TGC AGT CGA TGC GG
